# Dorsoventral patterning of the *Xenopus* eye involves differential temporal changes in the response of optic stalk and retinal progenitors to Hh signalling

**DOI:** 10.1186/s13064-015-0035-9

**Published:** 2015-03-20

**Authors:** Xiumei Wang, Giuseppe Lupo, Rongqiao He, Giuseppina Barsacchi, William A Harris, Ying Liu

**Affiliations:** The State Key Laboratory of Brain and Cognitive Science, Institute of Biophysics, Chinese Academy of Sciences, 15 Datun Road, Chaoyang District Beijing, 100101 China; Department of Physiology, Development and Neuroscience, University of Cambridge, Downing Street, Cambridge, CB2 3DY UK; Department of Chemistry, Sapienza University of Rome, Piazzale A. Moro 5, 00185 Rome, Italy; Dipartimento di Biologia, Unità di Biologia Cellulare e dello Sviluppo, Università di Pisa, SS 12 Abetone e Brennero 4, 56127 Pisa, Italy

**Keywords:** Optic vesicle, Optic stalk, Ventral retina, Dorsal retina, Hedgehog, *Xenopus*

## Abstract

**Background:**

Hedgehog (Hh) signals are instrumental to the dorsoventral patterning of the vertebrate eye, promoting optic stalk and ventral retinal fates and repressing dorsal retinal identity. There has been limited analysis, however, of the critical window during which Hh molecules control eye polarity and of the temporal changes in the responsiveness of eye cells to these signals.

**Results:**

In this study, we used pharmacological and molecular tools to perform stage-specific manipulations of Hh signalling in the developing *Xenopus* eye. In gain-of-function experiments, most of the eye was sensitive to ventralization when the Hh pathway was activated starting from gastrula/neurula stages. During optic vesicle stages, the dorsal eye became resistant to Hh-dependent ventralization, but this pathway could partially upregulate optic stalk markers within the retina. In loss-of-function assays, inhibition of Hh signalling starting from neurula stages caused expansion of the dorsal retina at the expense of the ventral retina and the optic stalk, while the effects of Hh inhibition during optic vesicle stages were limited to the reduction of optic stalk size.

**Conclusions:**

Our results suggest the existence of two competence windows during which the Hh pathway differentially controls patterning of the eye region. In the first window, between the neural plate and the optic vesicle stages, Hh signalling exerts a global influence on eye dorsoventral polarity, contributing to the specification of optic stalk, ventral retina and dorsal retinal domains. In the second window, between optic vesicle and optic cup stages, this pathway plays a more limited role in the maintenance of the optic stalk domain. We speculate that this temporal regulation is important to coordinate dorsoventral patterning with morphogenesis and differentiation processes during eye development.

**Electronic supplementary material:**

The online version of this article (doi:10.1186/s13064-015-0035-9) contains supplementary material, which is available to authorized users.

## Background

Vertebrate eye development begins during gastrulation, when the eye-forming region, known as the eye field, is induced within the anterior neural plate by signals produced by the underlying mesendoderm and other surrounding tissues [1-3]. During neurulation, the initially continuous eye field is split in two bilateral domains that evaginate from the lateral forebrain walls to form the optic vesicles [4,5]. Subsequently, invagination of the optic vesicles gives rise to the optic cups, which differentiate into the retinal pigmented epithelium (RPE), the neural retina and the optic nerve [6-8].

During these morphogenetic processes, the ventral part of the optic vesicle forms the optic stalk (OS), which connects the eye with the forebrain and later differentiates into the glial cells of the optic nerve, while the dorsolateral regions of the optic vesicle form the neural and pigmented retina [6,7,9]. This dorsoventral (DV) regionalization involves the differential expression of transcription factor encoding genes that subdivide the eye primordium in at least three domains: the ventrally located OS, expressing *Pax2*, *Vax1* and *Vax2*; the presumptive ventral retina (VR), expressing *Vax2* and the presumptive dorsal retina (DR), expressing *Tbx3* and *Tbx5* [10-12]. Similar to the spinal cord, regulation of gene expression along the eye DV axis depends on the ventralizing influence of hedgehog (Hh) ligands secreted from midline tissues (rostral mesendoderm and ventral forebrain) and the dorsalizing activity of bone morphogenetic protein (BMP)-like signals (GDF6 and BMP4) secreted from the dorsal pole of the eye bud and adjacent non-neural ectoderm [13-16].

While studies in zebrafish, *Xenopus*, chick and mouse embryos have all shown a role for Hh signalling in the ventralization of the eye [12,17-19], the developmental window during which the Hh pathway controls eye DV patterning and the temporal changes in the response of eye cells to Hh signals have been only partially investigated. In particular, there is a lack of information on the function of Hh signalling during the transition between eye field and optic vesicle stages, which is likely to be a critical window in the establishment of DV polarity, based on the dynamic expression patterns shown by relevant transcription factors [11]. In this study, we therefore used various molecular tools to activate or inhibit Hh signalling during specific stages of *Xenopus* eye development and we assessed the consequences of these manipulations on the DV polarity of the eye. All the employed experimental approaches suggest that the Hh pathway controls global DV patterning of the eye region, contributing to the specification of OS, VR and DR domains, as early as gastrula/neurula embryonic stages. Concomitantly with the emergence of the optic vesicle, the influence of Hh signalling on DR and VR fates decreases, as shown by increased resistance of the DR to Hh-dependent ventralization and by Hh-independent maintenance of VR fates. In contrast, Hh signalling continues to support OS gene expression during optic vesicle stages and this prolonged regulatory input is required for the maintenance of proper OS size.

## Results

### Upregulation of smoothened-dependent signalling in the developing *Xenopus* eye causes stage-dependent effects on ocular DV patterning

Several studies have shown that Hh signalling plays a crucial role in the specification of ventral ocular fates and that overexpression of this pathway in the developing eye causes ventralization of the dorsal eye region [10,12,13,17,18,20]. To gain insight into the developmental window during which upregulation of Hh signalling can affect eye DV polarity, we took advantage of purmorphamine (PMP), a small molecule agonist of smoothened (Smo), which was previously shown to activate the Hh pathway in the developing *Xenopus* eye [21]. *Xenopus* embryos were treated with 300 to 600 μM PMP starting from different stages, and the effects on eye DV patterning were scored at early optic cup stages (st. 33) by whole mount *in situ* hybridization. The following molecular markers were used as a readout for changes in the eye DV organization of treated embryos: *Pax2* and *Vax1b*, which are markers of the OS, the most ventral eye structure; *Vax2*, which is expressed both in the OS and in the overlying VR and *Tbx3*, a DR marker [11,12].

These experiments showed that activation of Hh signalling caused two different, temporally distinct, effects on the DV patterning of the eye. The stronger, and earlier, effect was the ventralization of the dorsal eye region, with upregulation of ventral eye markers throughout the eye and downregulation of DR markers. The weaker, and later, effect was the expansion of OS marker expression within the ventral, nasal and central retina, while expression of DR markers remained relatively unaffected. To quantify these different effects, we assigned an increasing score to PMP-treated embryos hybridized with probes for the OS markers *Pax2* and *Vax1b* depending on the extent of their ectopic expression into the dorsal half of the eye. Embryos in which transcription of OS markers remained confined within the ventral eye were given a 0 score. Embryos with limited, discontinuous upregulation of these genes into the dorsal eye, usually restricted to the dorsal marginal zone or to small groups of cells scattered in the dorsal eye, were scored as 1. Score 2 was assigned to embryos where expression of OS markers continuously spread from the ventral to the dorsal eye, but a substantial *Pax2/Vax1b*-negative dorsal domain persisted. Finally, embryos with a 3 score were those where *Pax2* or *Vax1b* expression covered most of the eye. This analysis was finished off by quantifying the fractions of embryos where expression of the VR marker *Vax2* spread through most of the eye and that of *Tbx3* was strongly reduced, while the rest of the embryos retained substantial *Vax2*-negative and *Tbx3*-positive domains within the dorsal eye.

**Figure 1:**
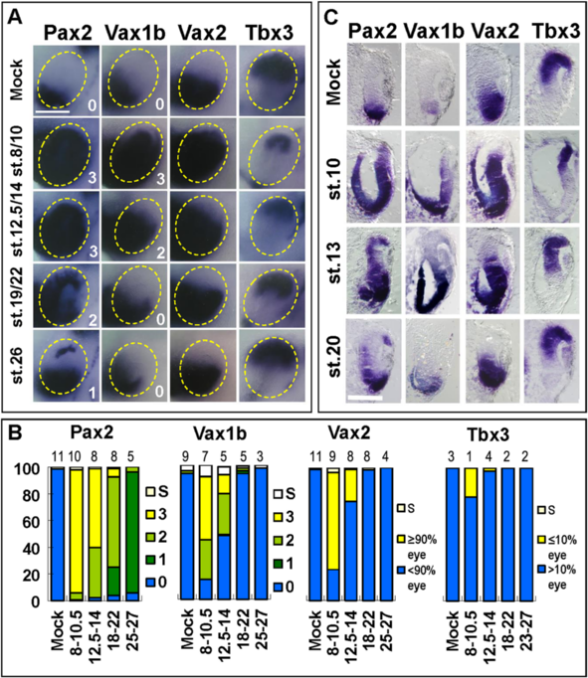
**PMP treatments cause stage dependent effects on eye DV polarity. (A)** Lateral views of heads of st. 33 embryos treated with DMSO (mock) or 300 to 600 μM PMP from the indicated stages and hybridized with probes for *Pax2*, *Vax1b*, *Vax2* or *Tbx3*. In mock-treated embryos, wild-type expression patterns of these genes are detectable: *Pax2* and *Vax1b* staining is restricted to the OS, *Vax2*-positive region covers the OS and VR, *Tbx3* expression identifies the DR. PMP treatments affect gene expression domains to various degrees depending on the stage of delivery (st. 8 to 10, blastula-early gastrula; st. 12.5 to 14, late gastrula-early neurula; st. 19 to 22, late neurula-early optic vesicle; st. 26, mid optic vesicle). For *Pax2* and *Vax1b*, embryos were grouped into 0 to 3 scores (numbers at the bottom right corner of each image) as explained in the ‘Results’ section and representative eyes for each score group are shown. See text for details. The broken yellow circles highlight the eye region. Scale bar, 200 μm. **(B)** Quantification of the percentages of embryos stained for *Pax2* or *Vax1b* with 0 to 3 scores in each treatment condition. Embryos stained for *Vax2* or *Tbx3* were grouped according to the DV extent of *Vax2/Tbx3* expression domain (more or less than 90% of the eye for *Vax2*; more or less than 10% of the eye for *Tbx3*). The percentages of embryos with strong eye reductions are also indicated (S, small eyes). The number of experiments performed for each probe and treatment condition is indicated on top of the corresponding histogram bar. At least 20 eyes/10 embryos were analysed for each experiment. **(C)** Histological sections of eyes of st. 33 embryos treated as in **(A)** and **(B)**, and hybridized with the indicated probes, confirming stage dependent alterations in the expression domains of *Pax2*, *Vax1b*, *Vax2* and *Tbx3* as detected in whole mount views. Scale bar, 100 μm.

PMP treatments started from late blastula/early gastrula stages (st. 8/10.5, Figure 1A,B,C) reproduced the effects of Hh ligand mRNA injections in early embryos [12], as they could cause upregulation of *Pax2*, *Vax1b* and *Vax2* throughout the eye and downregulation of *Tbx3*. In these conditions, the majority of treated embryos upregulated *Vax2* through most of the eye (73%) and a similar situation was found also for *Pax2* (score 3, 92%). Significant numbers of embryos also expressed *Vax1b* in a broad DV domain (score 3, 47%) or showed severely reduced expression of *Tbx3* (22%). The percentage of embryos with strong downregulation of *Tbx3* increased when PMP treatments were started from early cleavage stages (st. 4, Additional file 1: Figure S1). When PMP was delivered from early neurula stages (st. 12.5 to 14, Figure 1A,B,C), considerable numbers of embryos with strong expansion of the *Pax2* domain were still detectable (score 3, 59%), but the fractions of embryos with a broad spread of *Vax2* (24%) or *Vax1b* (score 3, 14%) were reduced and those with severe *Tbx3* reduction were nearly absent (3%). These conditions also resulted in significant numbers of embryos with partial (score 2) dorsal expansion of *Pax2* and *Vax1b*, so that nearly all of the *Pax2*-stained embryos (97%) and roughly half of the *Vax1b*-stained embryos (45%) were scored as 2 or 3. No embryos with *Vax2* expression covering most of the eye or strongly reduced *Tbx3* domain were found following treatments with PMP from late neurula/early optic vesicle stages (st. 18 to 22, Figure 1A,B,C), and a substantial DR domain was specified in these conditions. *Pax2*, however, was still markedly upregulated by these later treatments. Although *Pax2* expression seldom spread through most of the eye (score 3, 6%), almost all the embryos showed a partial dorsal expansion (score 1 or 2, 89%). *Vax1b* expression domain was also expanded, albeit only within the ventral half of the eye. Finally, no evident effects on *Vax2*, *Vax1b* or *Tbx3* were observed when PMP was applied from mid optic vesicle stages (st. 25 to 27, Figure 1A,B,C). In contrast, most of the embryos stained with *Pax2* probes showed restricted ectopic transcription at the level of the dorsal marginal zone (score 1, 90%). Double staining with *Tbx3* probes and an anti-Pax2 antibody confirmed that within the most dorsal retina, PMP treatments started during optic vesicle stages caused localized Pax2 protein expression in the dorsal marginal zone, which partially overlapped with *Tbx3* expression (Additional file 2: Figure S2).

**Figure 2:**
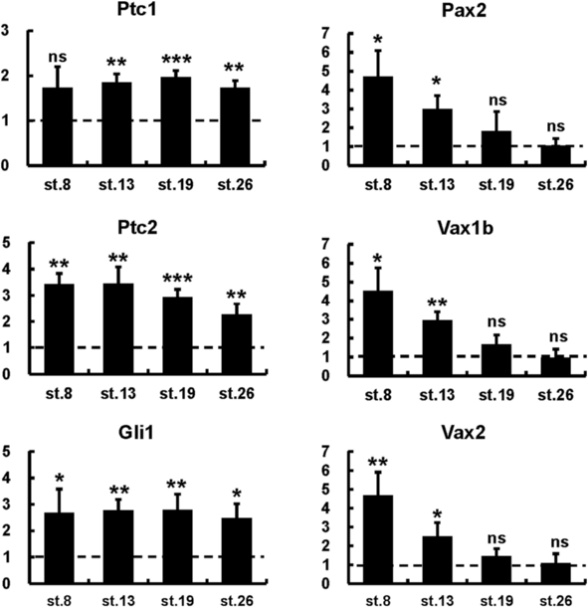
**Quantification of gene expression changes following PMP treatments.** Real-time PCR quantification of gene expression in st. 33 dissected heads following treatments with 600 μM PMP or DMSO from the indicated stages, shown as the mean ratio between PMP and DMSO conditions in four independent experiments. Error bars show standard deviations. *P < 0.05; **P < 0.01; ***P < 0.001; ns, non-significant (*P* ≥ 0.05) according to two-tailed Student’s *t*-test.

These temporally dependent effects were further characterized by real-time PCR analysis on dissected heads from control and PMP-treated embryos. The Hh pathway direct target genes *Ptc1*, *Ptc2* and *Gli1* were significantly upregulated following any PMP treatment condition, suggesting that the different effects of early and late treatments were not due to decreased efficiency of Hh signalling activation (Figure 2). In contrast, *Pax2*, *Vax1b* and *Vax2* were significantly upregulated by treatments started at st. 8 or st. 13, but not later (Figure 2), confirming that strong ventralization of the dorsal eye requires increased Hh signalling as early as gastrula/neurula stages.

**Figure 3:**
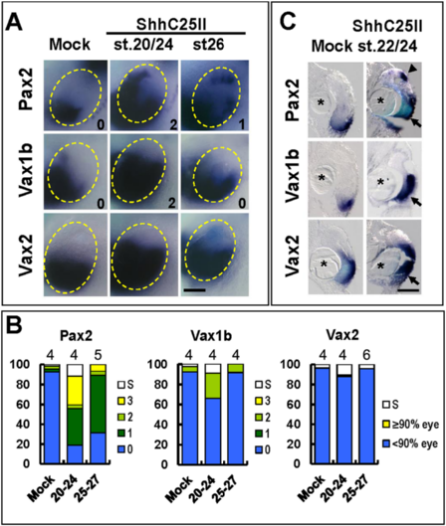
**Grafts of ShhC25II-soaked beads reproduce the stage-dependent effects of PMP treatments on eye DV patterning. (A)** Lateral views of heads of st. 33 embryos that received a graft of control or ShhC25II-soaked beads next to the optic vesicle at the indicated stages and hybridized with probes for *Pax2*, *Vax1b* or *Vax2*. Compared to controls, embryos grafted with ShhC25II beads show stage-dependent increase in the expression domains of ventral eye genes. Scale bar, 100 μm. **(B)** Quantification of the percentages of embryos with different effects on gene expression domains or eye reductions (S) in each treatment condition. The number of experiments performed for each probe and treatment condition is indicated on top of the corresponding histogram bar. **(C)** Histological sections of eyes of st. 33 embryos treated as in (A) and (B) and hybridized with the indicated probes, confirming stage dependent effects on DV eye patterning as detected in whole mount views. Triangles point to ectopic *Pax2* expression in the dorsal marginal zone and arrows to expanded ventral expression domains of *Pax2*, *Vax1b* and *Vax2*, in embryos grafted ShhC25II beads. Stars indicate the position of the beads. Scale bar, 100 μm.

This temporal analysis was validated by upregulating Hh signalling in the eye region by means of grafts of beads soaked in sonic hedgehog (C25II) N-terminus (ShhC25II), a bioactive form of the sonic hedgehog (Shh) protein [22]. ShhC25II beads grafted during early (st. 20 to 24) or mid (st. 25 to 27) optic vesicle stages reproduced the effects of PMP treatments during corresponding developmental windows (Figure 3A,B,C). In either case, a significant part of the dorsal eye remained devoid of expression of the VR marker *Vax2* in most of the embryos. Following early optic vesicle grafts, however, partial dorsal upregulation of OS markers was clearly detectable, especially along the nasal region (*Pax2*, score 2 to 3, 70%; *Vax1b*, score 2, 25%). Compared to PMP, ShhC25II beads resulted in some embryos expressing *Pax2* within a broad DV domain (score 3, 23%) and *Vax1b* was also more strongly upregulated by ShhC25II at these stages of treatment (compare *Pax2* and *Vax1b* charts in Figures 1B and 2B), suggesting that PMP treatments cause somewhat slower upregulation of the Hh pathway compared to grafts of ShhC25II beads. Grafts performed at mid optic vesicle stages (st. 25 to 27) yielded similar results to PMP treatments, as *Vax2* and *Vax1b* expression domains remained confined to the ventral eye region, while *Pax2* was ectopically expressed in the dorsal marginal zone.

Taken together, these results suggest that the Hh pathway plays temporally distinct functions during the specification of the DV polarity of the eye. During gastrula/neurula stages, Hh signalling may influence DV patterning throughout the eye region, as both the ventral and the dorsal eye are sensitive to ventralization by this pathway. By early optic vesicle stages, the competence of the DR to ventralization by Hh signalling appears to be markedly reduced, but this pathway can still increase OS marker expression in the ventral/central retina and in selected areas of the dorsal/nasal retina. Thus, at these later stages, Hh signalling may play a more restricted role in promoting OS fates within the ventral eye region.

### Temporally controlled overexpression of active Gli constructs reveals prolonged sensitivity of OS genes to Gli-dependent signalling

**Figure 4:**
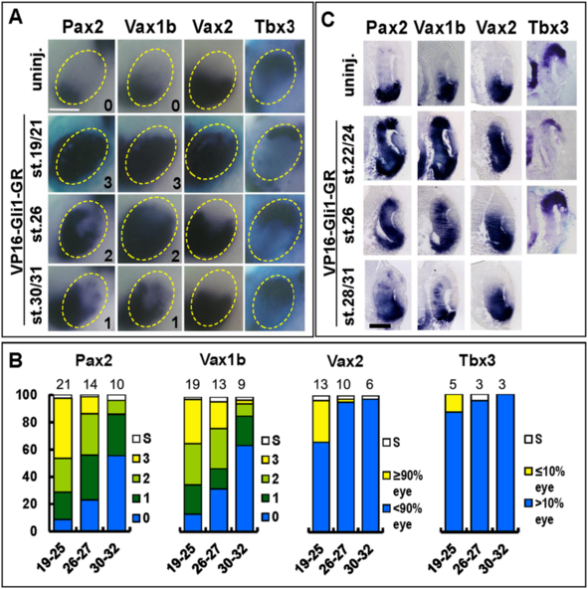
**Overexpression of** ***VP16-Gli1-GR*** **causes stage dependent effects on eye DV polarity. (A)** Lateral views of heads of st. 33 embryos that were unilaterally injected with 250 pg of *VP16-Gli1-GR* mRNA at the eight-cell stage, treated with dex from the indicated stages and hybridized with probes for *Pax2*, *Vax1b*, *Vax2* and *Tbx3*. Compared to the control side (uninj.), stage-dependent alterations in gene expression domains are detectable on the injected side. Light-blue β-gal staining identifies the injected side. Scale bar, 200 μm. **(B)** Quantification of the percentages of embryos with different effects on gene expression domains or eye reductions (S) in each treatment condition. The number of experiments performed for each probe and treatment condition is indicated on top of the corresponding histogram bar. **(C)** Histological sections of eyes of st. 33 embryos treated as in **(A)** and **(B)**, and hybridized with the indicated probes, confirming stage dependent gene expression changes as detected in whole mount views. Scale bar, 100 μm.

While both PMP treatments and grafts of ShhC25II-soaked beads caused effective activation of the Hh pathway in the eye, these reagents are likely to affect eye gene expression with some delay after the start of treatment, due to the time needed to build up sufficient amounts of ligands in the ocular extra-cellular space and to activate signal transduction within eye cells. Therefore, to dissect more precisely the time-dependent functions of Hh signalling in eye DV patterning, we employed a previously described *Gli1* chimeric construct, encoding for a fusion protein of the Gli1 DNA binding domain with the strong transcriptional activator domain of VP16 and the dexamethasone (dex)-inducible glucocorticoid receptor domain (*VP16-Gli1-GR*) [23]. In this case, *VP16-Gli1-GR* mRNA microinjections in early embryos allow accumulation of the fusion protein in eye cells, which can be promptly activated by exposing embryos to dex, leading to rapid transcription of target genes [23,24]. *VP16-Gli1-GR* mRNA was unilaterally injected into one dorsal animal blastomere at the eight-cell stage, and injected embryos were raised to the desired stage for dex treatment. No effects on eye development or molecular marker expression were seen in injected embryos in the absence of dex, while dex delivery during gastrula or early neurula stages caused strong eye reduction phenotypes (data not shown). Dex treatments from late neurula/early optic vesicle stages (st. 19 to 25, Figure 4A,B,C) did not decrease eye size, but caused partial ventralization of the dorsal eye (Figure 4A,B,C), as shown by significant fractions of embryos where most of the eye expressed *Vax2* (30%) or OS markers (*Pax2*, score 3, 44%; *Vax1b*, score 3, 33%) and by moderate reduction of *Tbx3* expression domain. Double staining for Pax2 protein and *Tbx3* mRNA expression showed a partial overlap between Pax2 and *Tbx3* staining, indicating that the ectopic Pax2-positive domain extended within the remaining *Tbx3*-expressing region (Additional file 3: Figure S3). When dex was added starting from mid optic vesicle stages (st. 26 to 27, Figure 4A,B,C), few embryos were found where most of the eye was positive for *Vax2* (2%) or for OS markers (*Pax2*, score 3, 12%; *Vax1b*, score 3, 20%), while the expression domain of *Tbx3* was largely unaffected, thus indicating that DR specification was mostly refractory to ventralization by Gli-mediated signalling by these stages. OS markers, however, were still significantly upregulated by these later treatments, and their expression domains were expanded in the ventral and central retina and, more dorsally, along the nasal part of the retina and/or in small groups of cells scattered in the dorsal half of the eye (*Pax2*, score 1 to 2, 63%; *Vax1b*, score 1 to 2, 45%). Finally, when dex treatments were started at the late optic vesicle/early optic cup stage (st. 30 to 32, Figure 4A,B,C), activation of Gli-mediated signalling could still promote ectopic expression of *Pax2* and *Vax1b* in scattered groups of cells within the central/dorsal retina (*Pax2*, score 1 to 2, 40%; *Vax1b*, score 1 to 2, 31%). Overall, the effects of *VP16-Gli1-GR* overexpression were similar to those of PMP treatments and ShhC25II bead grafts, but eye cells remained sensitive to ventralization by *VP16-Gli1-GR* for longer time windows compared to PMP or ShhC25II treatments. In particular, *VP16-Gli1-GR* overexpression from late neurula/early optic vesicle stages caused similar effects to PMP treatments from early neurula stages. Likewise, the effects of *VP16-Gli1-GR* overexpression from mid or late optic vesicle stages were similar to those of PMP/ShhC25II treatments from early or mid optic vesicle stages, respectively.

**Figure 5:**
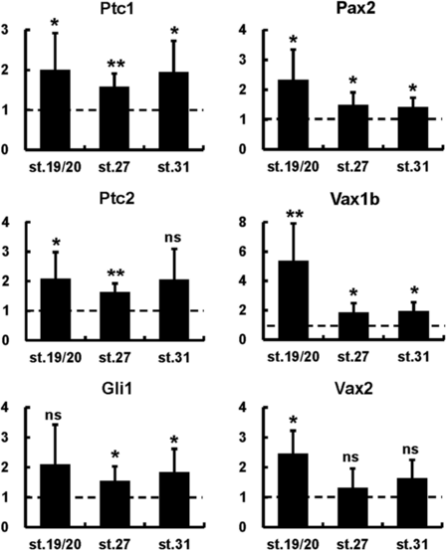
**Quantification of gene expression changes following** ***VP16-Gli1-GR*** **overexpression.** Real-time PCR quantification of gene expression in st. 33 heads dissected from controls or from embryos injected with *VP16-Gli1-GR* mRNA and treated with dex from the indicated stages. Results are shown as the mean ratio between *VP16-Gli1-GR*-injected and control embryos in six independent experiments. Error bars show standard deviations. *P < 0.05; **P < 0.01; ns, non-significant (*P* ≥ 0.05) according to two-tailed Student’s *t*-test.

In these experiments, real-time PCR analysis of gene expression showed that the Hh pathway direct target genes *Ptc1*, *Ptc2* and *Gli1* were significantly upregulated by *VP16-Gli1-GR* overexpression independently of the stage of dex treatment (Figure 5). In contrast, *Vax2* was significantly upregulated by dex treatments started from late neurula/early optic vesicle stages, but not later (Figure 5). Notably, we detected a significant increase in the expression levels of the OS markers *Pax2* and *Vax1b* in all treatment conditions, although the extent of their upregulation decreased in embryos treated from mid or late optic vesicle stages (Figure 5). Taken together, these results suggest that DR cells remain competent to ventralization by Gli-mediated signalling throughout neurula stages, while activation of this pathway can influence expression of OS genes within a more restricted eye region up to the initial stages of optic cup formation.

### Hh signalling is required for proper OS and VR specification during neurula stages and for maintenance of OS size during optic vesicle stages

We next investigated the requirement of functional Hh pathway during neurula and optic vesicle stages for proper eye DV patterning by loss-of-function experiments. To this aim, we downregulated Hh signalling during different time windows by treating embryos with the Smo antagonist cyclopamine (CPM) starting from progressively later developmental stages. Control and CPM-treated embryos were then harvested at st. 33, and the effects on the expression pattern of ventral and dorsal eye markers were assessed by *in situ* hybridization. We previously showed that CPM can effectively inhibit the Hh pathway in the developing *Xenopus* eye [12,25]. Moreover, real-time PCR analysis demonstrated that the Hh responsive genes *Ptc1*, *Ptc2* and *Gli1* were strongly downregulated by CPM irrespectively of the initial stage of treatment (data not shown).

**Figure 6:**
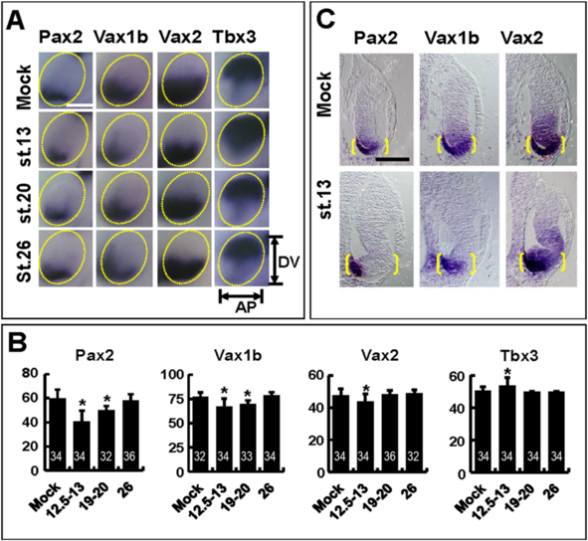
**CPM treatments cause stage dependent effects on eye DV polarity. (A)** Lateral views of heads of st. 33 embryos treated with ethanol (mock) or 100 μM CPM from the indicated stages and hybridized with probes for *Pax2*, *Vax1b*, *Vax2* or *Tbx3*. Compared to controls, CPM-treated embryos show stage-dependent changes in gene expression domains along the anteroposterior (AP; *Pax2*, *Vax1b*) or the DV (*Vax2*, *Tbx3*) axes of the eye. Scale bar, 200 μm. **(B)** Quantification of the mean AP (*Pax2*, *Vax1b*) or DV (*Vax2*, *Tbx3*) width/height of gene expression domains, normalized to total width/height of the eye, in the eyes of embryos treated as in **(A)**. The number of eyes analysed for each probe and treatment condition is indicated within the corresponding histogram bar. Error bars show standard deviations. *P < 0.05; ns, non-significant (*P* ≥ 0.05) according to two-tailed Student’s *t*-test. **(C)** Histological sections of eyes of st. 33 embryos treated with ethanol or CPM from st. 13 and hybridized with the indicated probes. CPM-treated eyes show a reduction of *Pax2*, *Vax1b* and *Vax2* expression domains along the eye proximodistal axis. Yellow brackets highlight the proximodistal extent of the whole ventral eye region. Scale bar, 100 μm.

When embryos were treated with CPM starting from late gastrula/early neurula stages (st. 12.5 to 13 Figure 6A,B,C), changes to the overall DV organization of the eye were detectable. *Vax2* expression domain in the VR was reduced along the eye DV axis, while *Tbx3* domain in the DR was expanded ventrally (Figure 6A,B). In addition, the ventrally located OS, marked by *Pax2* and *Vax1b* expression, was shortened (Figure 6A,B). Furthermore, transverse sections of embryos following whole-mount *in situ* hybridization revealed a proximo-distal reduction of *Pax2*, *Vax1b* and *Vax2* expression domains in the ventral eye region of CPM-treated embryos (Figure 6C). CPM delivery from late neurula/early optic vesicle stages (st. 19 to 20) did not affect the expression domains of *Vax2* and *Tbx3* in the VR and the DR, respectively (Figure 6A,B). Notably, OS size was still reduced by these later treatments (Figure 6A,B). Finally, no changes on eye DV patterning were detected when CPM was applied starting from mid optic vesicle stages (stage 26, Figure 6A,B). These results are consistent with those from overexpression assays and, together, they support a model where Hh signalling controls the specification of both the VR and OS during neurula stages and supports maintenance of OS size during optic vesicle stages.

## Discussion

**Figure 7:**
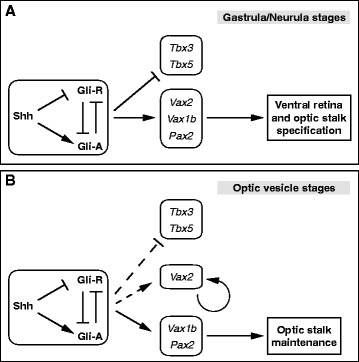
**Proposed model of the stage dependent effects of Hh signalling in the DV patterning of the** ***Xenopus*** **eye. (A)** During gastrula/neurula stages of development, Hh signalling controls the specification of both the OS and VR domains by promoting expression of OS (*Pax2*, *Vax1b*) and VR (*Vax2*) genes and repressing expression of DR genes (*Tbx3*, *Tbx5*). **(B)** During optic vesicle stages, the expression domains of *Vax2* and *Tbx3/5* become less dependent on Hh signalling (dashed lines), which is required to maintain *Pax2* and *Vax1b* expression and proper OS development. At both stages, activation of Hh signalling is likely to depend on the Shh ligand, acting through inhibition of Gli repressor proteins (Gli-R) and increase of Gli activator proteins (Gli-A) [40]. Vax2 was previously shown to self-activate its own expression at optic vesicle stages [29].

Hh signalling has a pivotal role in the specification of ventral positional identities in the eye. Overexpression experiments in zebrafish, *Xenopus* and chick embryos all caused expansion of OS and/or VR at the expense of DR [12,17,18,20], while zebrafish and mouse mutants for the Hh ligand Shh or the Hh pahway component Smo showed loss of ventral eye structures [13,18,19]. Here, we show that Hh signalling differentially controls eye DV patterning during two distinct temporal windows, one from the neurula to the optic vesicle stages and the other from the optic vesicle to the optic cup stages, respectively (Figure 7). During the earlier window, most of the eye field region was competent to ventralization by the Hedgehog pathway. We noticed, however, that Hh signalling could upregulate ventral eye genes more efficiently than it could downregulate dorsal eye genes. For example, effective repression of dorsal genes required stronger treatment conditions than those sufficient to expand ventral gene expression domains. Moreover, ventralization of dorsal eye tissue in response to increased Hh signalling was often incomplete, as indicated by partial overlap of ectopic ventral gene expression with areas retaining expression of dorsal genes. These observations suggest that Hh signalling may regulate ventral and dorsal eye genes independently of each other. While ventral genes appear to be highly responsive to Hh pathway activation, repression of dorsal genes may involve additional mechanisms, such as interactions between Hh and other ventralizing signals [12] and/or inhibition of BMP signalling [14-16,26]. Following this earlier window, our results suggest that during formation of the optic vesicle, DR, VR and OS progenitors differentially modify their responsiveness to Hh signalling. In particular, the presumptive DR loses competence to Hh-dependent ventralization, VR progenitors maintain their fates in spite of reduced Hh signalling, but they retain competence to acquire OS fates in response to increased Hh signalling, and OS progenitors continue to rely on active Hh signalling to maintain their identity.

In this study, we compared the effects of manipulation of Hh signalling with different pharmacological and molecular tools. In particular, we took advantage of small molecule Smo agonists (PMP) or antagonists (CPM) to activate/inhibit Smo-mediated signal transduction during specific time windows. ShhC25II-soaked beads were used to confirm the specificity of these pharmacological approaches. Finally, previously described hormone-inducible *Gli1* constructs [23] were employed for controlled activation of Hh signalling downstream of Smo. Similar results were obtained following PMP treatments or grafts of ShhC25II-soaked beads close to the optic vesicle. However, the rate of diffusion of these reagents within the extra-cellular space, and the time needed for intra-cellular transduction of Smo activation, may potentially delay the window of effective upregulation of the Hh pathway compared to the onset of treatment. The previously characterized dex-inducible *Gli1* fusion construct (*VP16-Gli1-GR*), which directly activates Hh target genes following induction [23], appeared to act more rapidly. Yet, experiments with PMP, ShhC25II beads and *VP16-Gli1-GR* all led to congruent results, which were mirrored by loss-of-function experiments with CPM. The partial reduction of VR and/or OS gene expression that we observed following Smo antagonism is consistent with previous studies [12,25], where it was explained with the partially redundant roles of Hh, retinoic acid (RA) and fibroblast growth factor (FGF) pathways in the DV patterning of the *Xenopus* eye [12].

These results, showing differential temporal changes in eye cell competence based on DV position, suggest a possible mechanism to avoid that retinal DV patterning is perturbed by the later release of Hh signals by differentiating retinal ganglion cells (RGCs) and the RPE, which regulate the retinal differentiation programme by activating the Hh pathway within the inner retinal layers, in the marginal zone and in the RPE, at the level of both the dorsal and the ventral optic cup, as previously described [25,27,28]. On the other hand, prolonged reliance of OS gene expression on Hh signalling during optic vesicle stages may be associated with the dynamic expression patterns of OS genes during the transition from the eye field to the optic cup and to the relatively late emergence of a well-defined boundary between the OS and the retinal territories. During *Xenopus* eye development, for example, *Pax2* expression domain in the neural plate eye field largely overlaps with that of *Vax2*, extending well within the prospective ventral retina. The *Pax2*-positive eye region then progressively recedes towards the ventral midline, but it still embraces *Vax2*-positive presumptive ventral retina at early optic vesicle stages and only during optic cup formation its lateral limit becomes coincident with the boundary between the OS and the retina [11]. This dynamic, Hh-dependent regulation of OS gene expression may be important to coordinate positioning of the OS/retinal boundary with the complex morphogenetic processes forging eye field cells into the optic vesicle and eventually into the optic cup and stalk.

What could be the molecular mechanisms controlling the temporal changes in the response of retinal cells to Hh signalling? The fact that forced expression of active Gli constructs cannot ventralize the DR during mid/late optic vesicle stages, reproducing the effects of treatments with PMP/ShhC25II-soaked beads, rules out the possibility that these changes simply depend on DR cells becoming unable to transduce Smo-mediated signalling at later stages of eye development. This would also be inconsistent with the later roles of RGC- and RPE-derived Hh signals in retinal differentiation. Instead, we suggest that eye cells remain responsive to Hh signalling but modify the ability of this pathway to regulate DV patterning genes using both intrinsic and extrinsic mechanisms. For example, epigenetic mechanisms may modify access of Gli factors to VR and DR gene chromatin during optic vesicle stages. In addition, Vax2 ability to self-activate its own expression [29] could contribute to make ventral retinal identity independent of Hh signalling after its initial establishment. Furthermore, collaboration with RA and FGF pathways may increase sensitivity of eye field cells to Hh signalling during neurula stages [12], while increased expression of BMP-like molecules (GDF6, BMP4, and so on) within the dorsal optic vesicle is likely to provide an effective barrier against Hh-dependent ventralization at later stages [16]. Clearly, more work is needed to unravel the interplay between intrinsic and extrinsic mechanisms in regulating the response of eye cells to Hh signalling.

Temporal changes in the competence of the responding tissues have been shown to play key roles in Hh-dependent DV patterning in other neural tube locations, such as the spinal cord and telencephalon [30-33]. In these regions, however, Hh-dependent specification of the most ventral domain is restricted to an early temporal window, whereas, in the developing eye, the most ventral ocular fates (OS) show prolonged reliance on Hh signalling. In future work, it will be interesting to investigate whether eye cells carry out stage-dependent modifications in the responsiveness to Hh signalling using similar or distinct mechanisms to those acting at different levels of the neural tube.

## Conclusions

In *Xenopus*, Hh signalling controls the establishment of the DV polarity of the eye and the specification of OS, VR and DR domains during a developmental window mainly limited to gastrula/neurula stages. This window is defined by temporal changes in the competence of presumptive DR cells, which become refractory to Hh-dependent ventralization during optic vesicle stages. At these stages, however, Hh signalling continues to be required in the ventral eye region for the maintenance of the OS domain. In line with previous studies in the telencephalon and the spinal cord [30-33], these results suggest that coordination of neural tube patterning and morphogenesis relies on stage-dependent regulation of morphogen signalling.

## Methods

### *Xenopus* embryos and treatments with PMP, CPM or ShhC25II-soaked beads

Embryos were obtained from *Xenopus laevis* frogs (Nasco, Atlanta, GA, USA) by *in vitro* fertilization at facilities at the Institute of Biophysics (Beijing, China) and the University of Cambridge (Cambridge, UK), raised and staged as previously described [34]. For PMP treatments, a 60 mM stock solution of PMP (Calbiochem, San Diego, CA, USA) in DMSO was diluted to 300 to 600 μm in 0.1× Marc’s modified Ringer’s (MMR). For CPM treatments, a 10 mM stock solution of CPM (Sigma-Aldrich, St. Louis, MO, USA) in 100% ethanol was diluted to 100 μM in 0.1× MMR. Embryos were incubated in these solutions at 22°C in the dark and allowed to grow to the desired stage. Control sibling embryos were cultured in parallel to PMP- or CPM-treated embryos in 0.1× MMR containing equivalent concentrations of DMSO or ethanol. ShhC25II-soaked beads were prepared using heparin-coated acrylic beads (diameter <100 μM; Sigma-Aldrich, St. Louis, MO, USA), which were washed in phosphate buffer saline (PBS) and soaked overnight at 4°C in a 0.5 μg/μl solution of recombinant mouse Sonic hedgehog (C25II), N-terminus (ShhC25II; R&D Systems, Minneapolis, MN, USA) and 1 μg/μl bovine serum albumin (BSA) in sterile PBS. Control beads were soaked in a 1.5 μg/μl solution of BSA in PBS. Embryos were anaesthetized in 0.15% MS222 (Sigma-Aldrich, St. Louis, MO, USA) in 0.1× MMR, transferred to 0.1× MMR and grafted with ShhC25II or control beads through a small slit made on one side of the optical vesicle by sharp forceps. After healing, embryos were cultured in 0.1× MMR to the desired stage. All animal work was approved by the Animal Welfare and Biological Research Ethics Committee at the Institute of Biophysics, Chinese Academy of Sciences (reference number SYXK2014-30) and performed in accordance with the Guide for the Care and Use of Laboratory Animals of the National Institute of Health (China).

### RNA methods and microinjections

The *VP16-Gli1-GR* plasmid, encoding for a fusion protein of Gli1 DNA binding domain with the VP16 transcriptional activation domain and the glucocorticoid receptor (GR) ligand binding domain, was previously described [23]. Capped mRNAs were synthesized and microinjected into one dorsal animal blastomere of eight-cell stage embryos as previously described [35]. For identification of the injected side, embryos were co-injected with β-galactosidase mRNA and stained as previously described [12].

### Whole mount *in situ* hybridization and real-time PCR

For *in situ* hybridization, embryos were fixed in MEMFA and processed following previously published protocols [36]. Sectioning of whole mount hybridized embryos was carried out as previously described [11]. Double staining for Pax2 protein and *Tbx3* mRNA expression was performed on sections (10 μm) from paraffin embedded embryos by fluorescent *in situ* hybridization with *Tbx3* probes, followed by immunohistochemistry with an anti-Pax2 antibody (Abcam, Cambridge, UK) as second antibody, as previously described [37]. For real-time PCR analysis, total RNA was extracted from dissected heads of st. 33 embryos, retro-transcribed and amplified on a Rotor-Gene Q (Qiagen, Hilden, Germany) using Qiagen kits as previously described [38]. Relative gene expression levels in different samples were determined with the Relative Standard Curve Quantification method [39] using *H4* [1] as a normalizer. Primers for real-time PCR were designed using Primer3 (http://bioinfo.ut.ee/primer3/) and are available on request. Statistical analysis of experimental data was performed with Microsoft Excel software.

## Additional files

Additional file 1: Figure S1. PMP treatments from early cleavage stages repress *Tbx3* expression. (A-D) Lateral views of the whole embryos (A and B) and eyes (C and D) of representative st. 33 embryos that were treated with DMSO (mock) or 300 μM PMP from the 8-cell stage (st. 4) and hybridized with probes for *Tbx3*, showing that early PMP treatments can strongly downregulate *Tbx3* expression in the DR. Scale bars, 1 mm for (A) and (C), and 0.1 mm for (B) and (D). (E) Quantification of the percentages of embryos grouped according to the DV extent of *Tbx3* expression domain (more or less than 10% of the eye). S, small eyes. The number of embryos analysed for each treatment condition is indicated on top of the corresponding histogram bar. Additional file 2: Figure S2. Ectopic Pax2 protein expression in the dorsal marginal zone of PMP-treated embryos. Histological sections of eyes of st. 33 embryos that were treated with DMSO (mock) (A and D) or 300 μM PMP (E-H, E’-H’) from stage 20, and double-stained by fluorescent in situ hybridization with *Tbx3* probes (red signal), followed by immunohistochemistry with an anti-Pax2 antibody (green signal). Cell nuclei were stained with Hoechst (blue signal). PMP treatments started from optic vesicle stages cause localized ectopic expression of Pax2 protein at the level of the dorsal marginal zone, which partially overlaps with *Tbx3*-expression. Enlarged pictures (E’-H’) are higher magnification images of boxed areas in (E-H), corresponding to the dorsal eye. Scale bars, 100 μm for (A-H), and 50 μm for (E’-H’). Additional file 3: Figure S3. Ectopic Pax2 expression within *Tbx3*-positive dorsal eye regions following *VP16-Gli1-GR* overexpression. Histological sections of control (A-D) or injected (E-H, E’-H’) eyes of st. 33 embryos that were unilaterally injected with 250 pg of *VP16-Gli1-GR* mRNA at the eight-cell stage, treated with dex from st. 22, and double-stained for Pax2 protein (green signal) and *Tbx3* mRNA expression (red signal). Cell nuclei were stained with Hoechst (blue signal). Following VP16-Gli1-GR overexpression, ectopic Pax2 protein expression is detectable in the dorsal eye, where it partially overlaps with *Tbx3* expression. Enlarged pictures (E’-H’) are higher magnification images of boxed areas in (E-H), corresponding to the dorsal eye. Scale bar, 100 μm for (A-H), and 20 μm for (E’-H’).

## Abbreviations

AP: anteroposterior
BMP: bone morphogenetic protein
BSA: bovine serum albumin
CPM: cyclopamine
dex: dexamethasone
DR: dorsal retina
DV: dorsoventral
FGF: fibroblast growth factor
GR: glucocorticoid receptor
Hh: hedgehog
MMR: Marc’s modified Ringer’s
OS: optic stalk
PBS: phosphate buffer saline
PMP: purmorphamine
RA: retinoic acid
RGC: retinal ganglion cell
RPE: retinal pigmented epithelium
Shh: sonic hedgehog
ShhC25II: sonic hedgehog (C25II) N-terminus
Smo: smoothened
st.: stage
VR: ventral retina
