# Supplementary figures and images for: Dorsoventral patterning of the Xenopus eye involves differential temporal changes in the response of optic stalk and retinal progenitors to Hh signalling
Source: Neural Dev. 2015 Mar 20;10:7. doi: 10.1186/s13064-015-0035-9 (PMC4373414; doi:10.1186/s13064-015-0035-9)

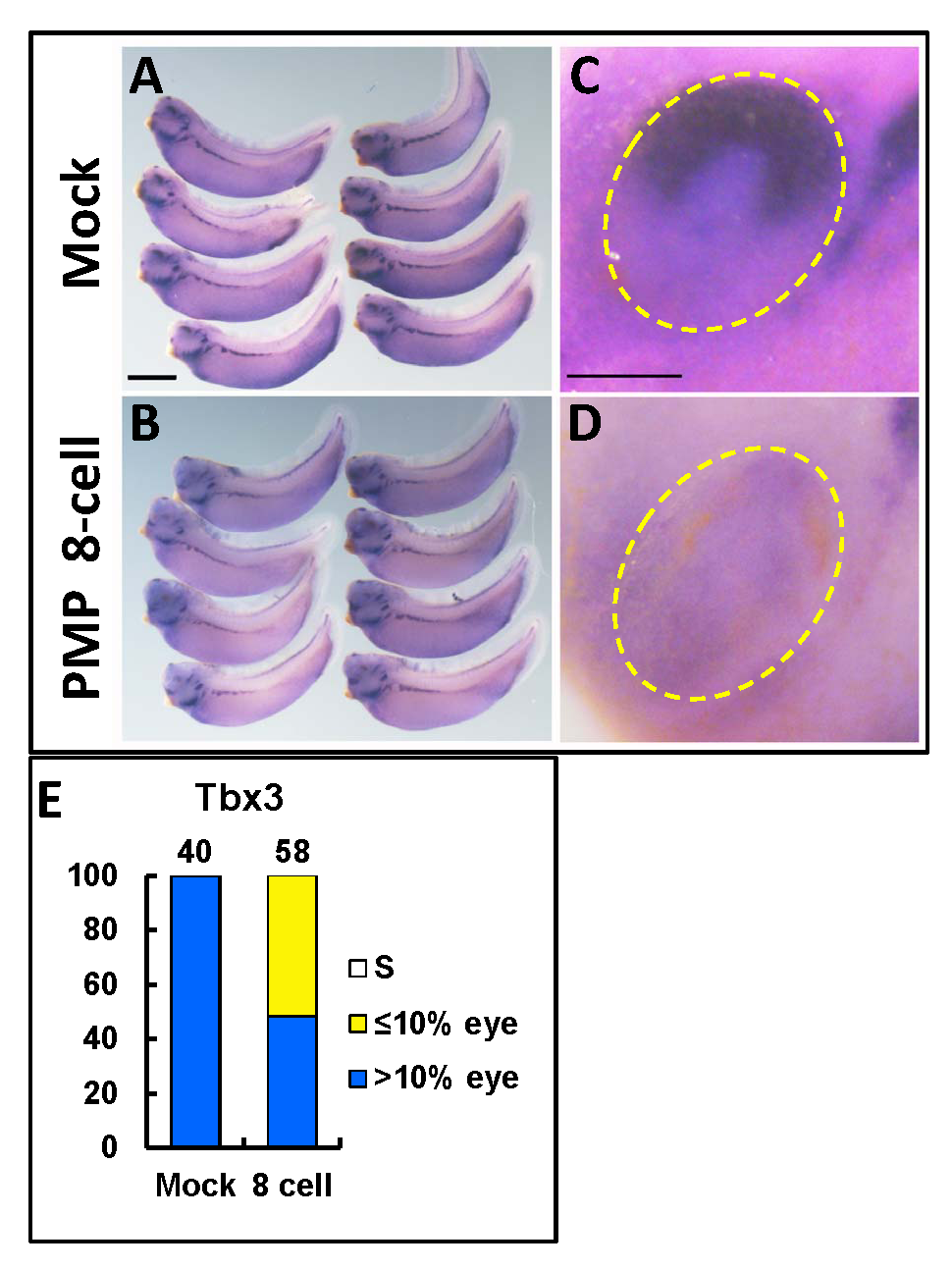

Supplement: Additional file 1: Figure S1. — PMP treatments from early cleavage stages repress Tbx3 expression. (A-D) Lateral views of the whole embryos (A and B) and eyes (C and D) of representative st. 33 embryos that were treated with DMSO (mock) or 300 μM PMP from the 8-cell stage (st. 4) and hybridized with probes for Tbx3, showing that early PMP treatments can strongly downregulate Tbx3 expression in the DR. Scale bars, 1 mm for (A) and (C), and 0.1 mm for (B) and (D). (E) Quantification of the percentages of embryos grouped according to the DV extent of Tbx3 expression domain (more or less than 10% of the eye). S, small eyes. The number of embryos analysed for each treatment condition is indicated on top of the corresponding histogram bar. [file 13064_2015_35_MOESM1_ESM.tiff]

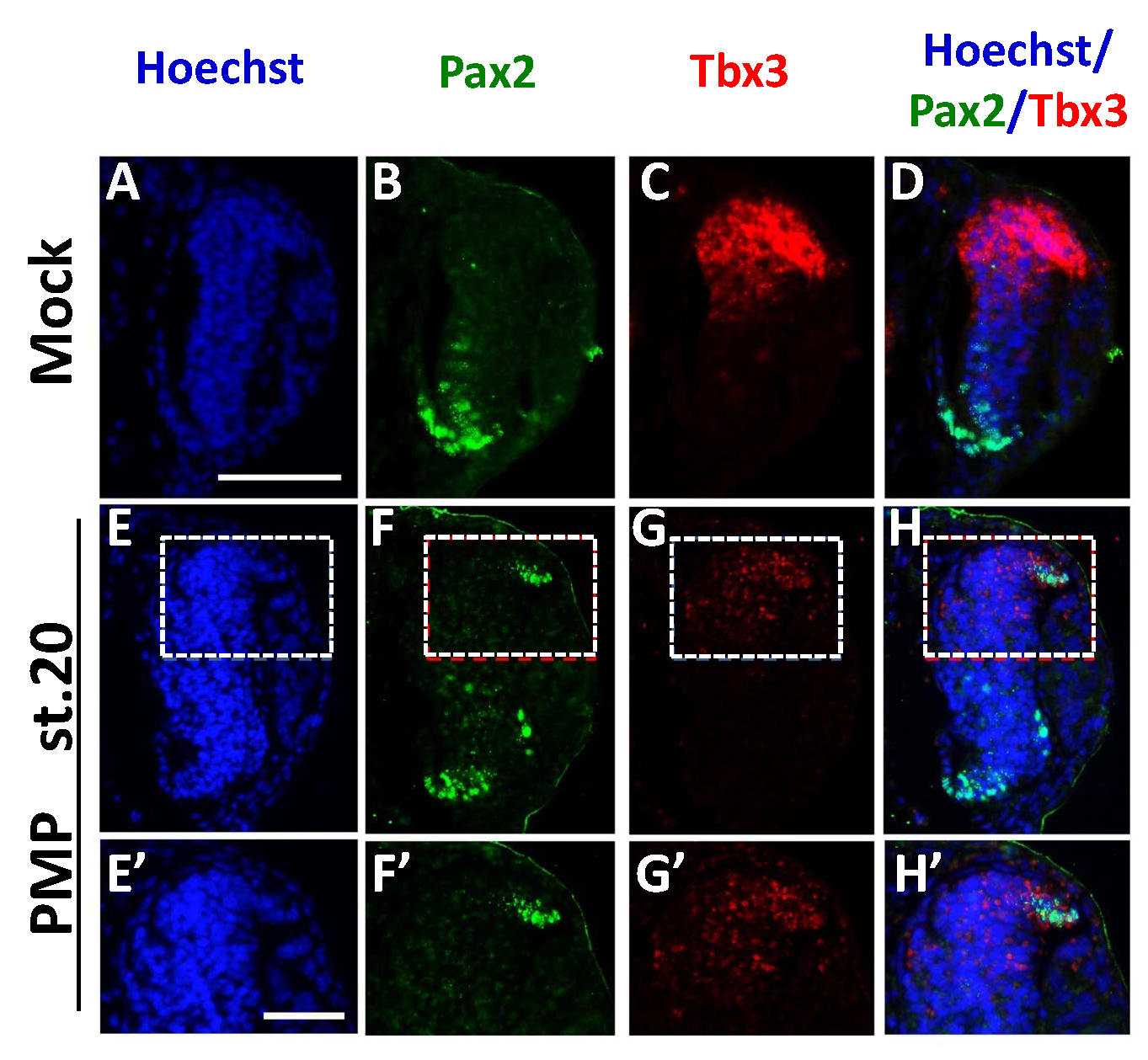

Supplement: Additional file 2: Figure S2. — Ectopic Pax2 protein expression in the dorsal marginal zone of PMP-treated embryos. Histological sections of eyes of st. 33 embryos that were treated with DMSO (mock) (A and D) or 300 μM PMP (E-H, E’-H’) from stage 20, and double-stained by fluorescent in situ hybridization with Tbx3 probes (red signal), followed by immunohistochemistry with an anti-Pax2 antibody (green signal). Cell nuclei were stained with Hoechst (blue signal). PMP treatments started from optic vesicle stages cause localized ectopic expression of Pax2 protein at the level of the dorsal marginal zone, which partially overlaps with Tbx3-expression. Enlarged pictures (E’-H’) are higher magnification images of boxed areas in (E-H), corresponding to the dorsal eye. Scale bars, 100 μm for (A-H), and 50 μm for (E’-H’). [file 13064_2015_35_MOESM2_ESM.tiff]

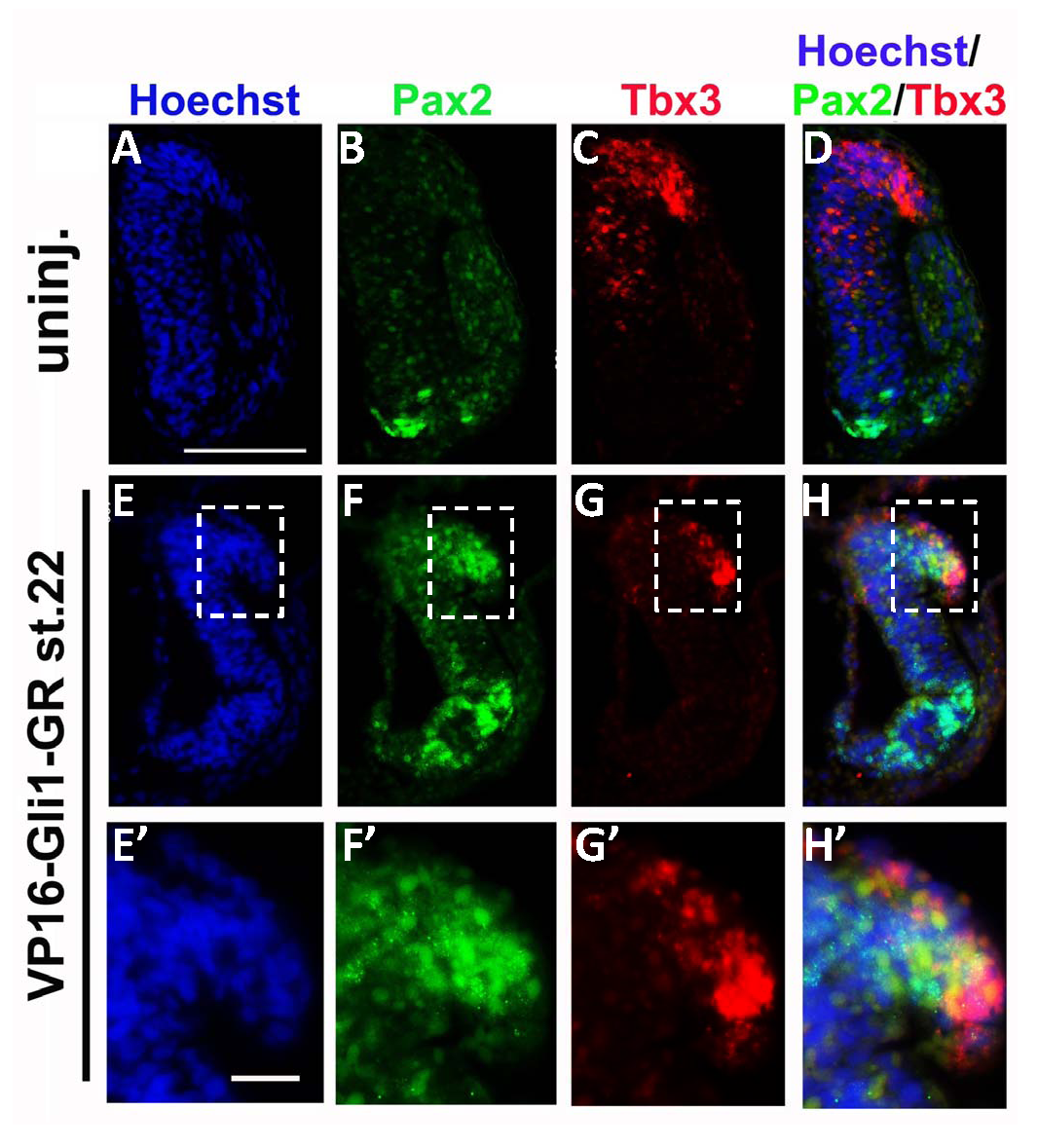

Supplement: Additional file 3: Figure S3. — Ectopic Pax2 expression within Tbx3-positive dorsal eye regions following VP16-Gli1-GR overexpression. Histological sections of control (A-D) or injected (E-H, E’-H’) eyes of st. 33 embryos that were unilaterally injected with 250 pg of VP16-Gli1-GR mRNA at the eight-cell stage, treated with dex from st. 22, and double-stained for Pax2 protein (green signal) and Tbx3 mRNA expression (red signal). Cell nuclei were stained with Hoechst (blue signal). Following VP16-Gli1-GR overexpression, ectopic Pax2 protein expression is detectable in the dorsal eye, where it partially overlaps with Tbx3 expression. Enlarged pictures (E’-H’) are higher magnification images of boxed areas in (E-H), corresponding to the dorsal eye. Scale bar, 100 μm for (A-H), and 20 μm for (E’-H’). [file 13064_2015_35_MOESM3_ESM.tiff]
